# Comparative anti-methanogenic ability of green algae (*C. reinhardtii*) with/without nanoparticles: *in vitro* gas and methane production

**DOI:** 10.3389/fvets.2025.1492230

**Published:** 2025-02-03

**Authors:** Valiollah Palangi, Adem Kaya, Muhlis Macit, Hayrunnisa Nadaroglu, Hayrullah Bora Ünlü, Ali Kaya, Ashkan Fekri, Ayaz Mammadov, Maximilian Lackner

**Affiliations:** ^1^Department of Animal Science, Faculty of Agriculture, Ege University, Izmir, Türkiye; ^2^Department of Animal Science, Faculty of Agriculture, Ataturk University, Erzurum, Türkiye; ^3^Department of Nano-Science and Nano-Engineering, Institute of Science and Technology, Ataturk University, Erzurum, Türkiye; ^4^Department of Animal Science, College of Agriculture and Natural Resources, University of Tehran, Alborz, Karaj, Iran; ^5^Department of Life Sciences, Western Caspian University, Baku, Azerbaijan; ^6^Department of Industrial Engineering, University of Applied Sciences Technikum Wien, Vienna, Austria

**Keywords:** gas production, nanoparticles, methane emission, *in vitro*, green algae

## Abstract

**Introduction:**

The purpose of this study was to investigate how in vitro gas production (GP) and ruminal fermentation characteristics were affected by increasing concentrations of green algae plant (*C. reinhardtii*) extracts in combination with nanoparticles MgO and MgS.

**Methods:**

A solution containing 0.1 M MgCl_2_ was prepared in 300 mL for the green production of MgCl nanoparticles. The mixture was refluxed for two hours at 85°C using a reflux condenser after 10 mL of pomegranate plant extract was added. The green algal plant (*C. reinhardtii*), which has many non-toxic antioxidants, was used as a carbon source to produce carbon quantum dots (CQD). Chemical analysis was conducted in accordance with AOAC (2005) recommendations. Rumen fluid from recently slaughtered calves is used to produce *in vitro* gas immediately following slaughter. Analysis of variance (ANOVA) was performed on the obtained data from the *in vitro* study in a completely randomized design using the mixed model of SAS (version 9.4; Inc., Cary NC, USA).

**Results and Discussion:**

The variance analysis results and the average values of the chemical compositions were significantly influenced by the extracts (all *p *< 0.0001). In this line, the values of net gas, pH, OMD, ME, NEl, and ME were found to be the highest for Algae + 50 MgO and the lowest for Algae + 50 MgS, respectively (all *p* < 0.0001). These promising results imply that extracts from *C. Reinhardtii* may be able to mitigate the adverse consequences of rumen fermentation. To precisely ascertain the impact particular Rhodophyta on greenhouse gas emissions, additional investigation is needed.

## Introduction

In response to the increasing population and the need to provide animal protein, along with the lack of animal feed resources, humans and animals have competed for agricultural resources (1, 2). Thus, Sustainability of livestock production is currently a research priority due to the increasing demand for food by the growing world population. It has been predicted that green algae (*Chlamydomonas reinhardtii*) can provide biomass and animal feed in the future (3). Green algae are substantially more productive in terms of biomass than other photosynthetic organisms, and more crucially, growing microalgae does not compete with food crops on arable ground (4). It is possible to use the algae as a non-traditional alternative feed source owing to their efficacy in converting solar energy, independence from external environmental conditions, and high production rate compared to conventional crops (4).

Besides contributing to greenhouse gas emissions, methane loss is one of the greatest negative factors in ruminant production (5–7). Although causing energy loss in the rumen, CH_4_ production reduces rumen acidity and keeps the rumen environment below normal via using H^+^ ions by methanogenic bacteria (8). The concentration of dihydrogen in the rumen depends on factors such as methanogen growth and the rate of feed fermentation. Methane generation and volatile fatty acid production are determined by the equilibrium between pathways that create and combine metabolic hydrogen (9). A variety of methane inhibitors can prevent methane-related energy losses in ruminants and provide economic and ecological benefits (10).

Numerous resources have focused on the reduction of CH_4_ generation, especially energy loss from methane production. In addition, studies on the transformation of fermentation products into chemicals useful for animals have been accompanied in the recent years. Accordingly, to reduce enteric methane production, unsaturated fatty acids (11, 12), lysozyme (13), organic acid salts (14), *S. cerevisiae* (15), enzymes (15), and ethyl acetate (16) are added to ruminant diets. Unlike specific CH_4_ inhibitors, these compounds generally affect and suppress microorganism growth (17). Consequently, the feed value is reduced due to adverse effects on rumen fermentation. Many researchers suggest that, instead of adding additives that are thought to affect the rumen microbiome, the use of carbon quantum dots (CQD), magnesium sulfide (MgS) and magnesium oxide (MgO) nanoparticles, which are known as hydrogen receptors, is an appropriate alternative (18–21). However, there is a lack of information about the evaluating anti-methanogenic capabilities of nanoparticles of *C. reinhardtii* in *in vitro* system. Thus, this study assessed, using an *in vitro* gas and methane generation approach, the green algal (*C. reinhardtii*) anti-methanogenic capabilities with and without nanoparticles.

## Materials and methods

### Green synthesis and structural characterization of CQD, MgS and MgO NPs

#### Preparation of algae extract

For the green synthesis of MgCl NPs, 300 mL of a solution containing 0.1 M MgCl_2_ was prepared. 10 mL of pomegranate algae extract was added to the solution and refluxed for 2 h at 85°C under a reflux condenser. It was then placed in a reactor via Teflon tube. Hydrothermal reactions were performed at 180–195°C for 4 h to reduce nano-particle (NP) size. The precipitated MgO NPs were washed first via pure water and ethyl alcohol. They were preserved in an atmosphere free of moisture after being dried for 48 h at 60°C in an oven. MgS NPs were synthesized using the same procedure. 1 mol of Na_2_S was added to the synthesis medium and the same process was repeated to synthesize MgS NPs. In the synthesis of CQD, the green algae (*C. reinhardtii*), known for its high non-toxic antioxidant content, was used as a carbon source. For this purpose, the algae extract was placed in a reactor containing sodium citrate as a reducing agent. CQD was synthesized by incubating at 180–195°C for 8 h.

#### Characterization of CQD, MgS and MgO NPs

Green-synthesised CQD, MgS and MgO NPs were characterized at the High Technology Application and Research Center of Eastern Anatolia (DAYTAM) at Atatürk University. X-ray microscopy (XRD) and FTIR analyses were performed for the characterization of CQD, MgS, and MgO NPs. The synthesized CQD, MgS, and MgO nanoparticles were characterized, including their size and morphology.

#### Chemical analyses

AOAC (71) guidelines were followed for chemical analyses. Kjeldahl was used to determine N content (AOAC, 71, Method 984.13). For the determination of Acid Detergent Fiber (ADF) and Neutral Detergent Fiber (NDF), Van Soest et al. (22) were used.

#### *In vitro* gas production

*In vitro* gas production is performed by taking rumen fluid from newly slaughtered cattle (as soon as they are slaughtered), as mentioned by Palangi et al. (10). Using a method validated by Menke and Steingass (23), it was found that 0.2 g of treated (CQD, MgS, and MgO nanoparticles at levels of 0.50, 100 ppm) and ground (1 mm) green algae (*C. reinhardtii*) samples were incubated in rumen fluid via 100 mL standardized glass syringes to measure *in vitro* gas production. Methane and gas volumes of feed samples were measured 24 h after incubation.

#### Statistical analysis

The mixed model of SAS version 9.4 (SAS Institute, Inc., Cary, NC, United States) was used in a completely randomized design to examine the data gathered from the *in vitro* study. The following model was used to statistically analyze the experiment:


$$Y_{ij}=\mu +T_{i}+E_{ij}\text{.}$$

where μ is the overall mean for each parameter, T_i_ is the effect of treatment, and E_ij_ is residual error. Differences among sample means with *p* < 0.05 were accepted as statistically significant.

## Results

### Characterization of CQD, MgS, and MgO nanoparticles

#### XRD analysis

**Figure 1:**
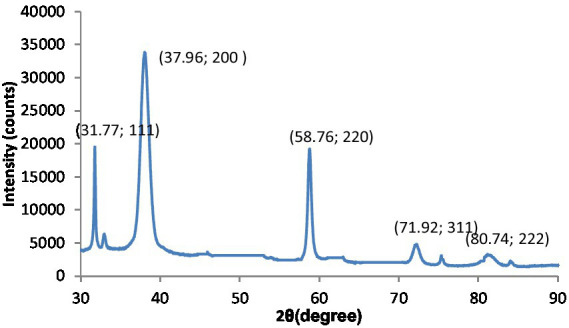
XRD patterns of MgO.

The fundamental method for examining crystal size, phase purity, and crystal structure is X-ray diffraction (XRD) examination. As shown in Figure 1, the XRD pattern of the synthesized MgO exhibits various peaks corresponding to the (111), (200), (220), (311), and (222) reflection planes.

The particle size of the synthesized MgO NPs was determined from the Debye–Scherrer equation: D = K/cos (*θ*).

The MgO NPs’ median dimension and d-spacing values have been determined to be 20 nm and 0.25 nm, correspondingly.

**Figure 2:**
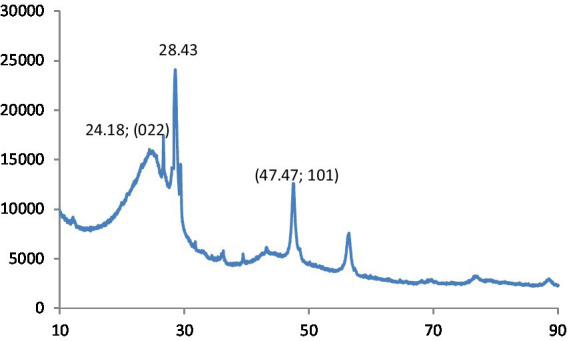
XRD patterns of CQD.

Figure 2 shows the XRD pattern of CNPs. The XRD pattern exhibited an intense peak at 2θ = 22.90° and a weak peak at 2θ = 41.60°, corresponding to the (022) and (101) diffraction patterns of graphite carbon, respectively.

#### FTIR analysis

**Figure 3:**
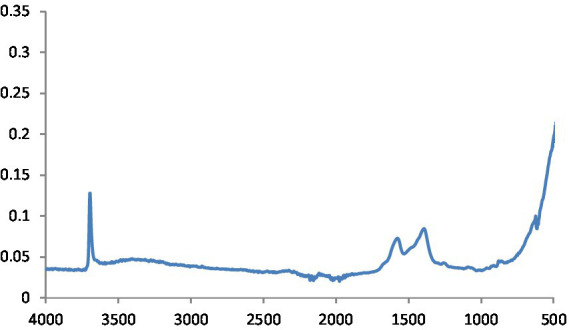
FTIR spectra of MgO NPs.

MgO NPs are characterized using the FTIR spectrum (Figure 3). In ambient settings, the spectra were captured at wavelengths ranging from 400 to 4,000 cm^−1^. The peak at 651.94 cm^−1^ indicates the stretching peak vibration of Mg-O bond, confirming that the obtained product is magnesium oxide. Moreover, H_2_O adsorption on the metal surface is indicated by the peaks at 1552.0 cm^−1^ and 3520.0 cm^−1^.

**Figure 4:**
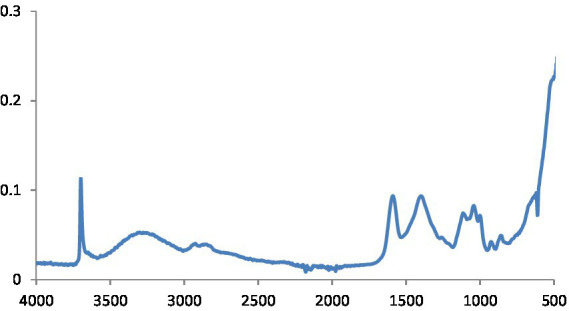
FTIR spectra of MgS NPs.

The potential biomolecules in charge of the reduction of MgS NPs by green synthesis were found using Fourier transform infrared spectroscopy (FTIR) analysis. The FTIR spectra of MgS NPs made using Na_2_S and pomegranate algae extract are displayed in Figure 4. In the spectrum, bands were observed at 3603.5, 1,725, 1,550, 1,232, 972 and 613 cm^−1^. Particularly, the sharp band at 1,725 cm^−1^ represents the C=O vibrations specific to the structure of flavonoids that can be found in pomegranate extract.

#### TEM analysis

**Figure 5:**
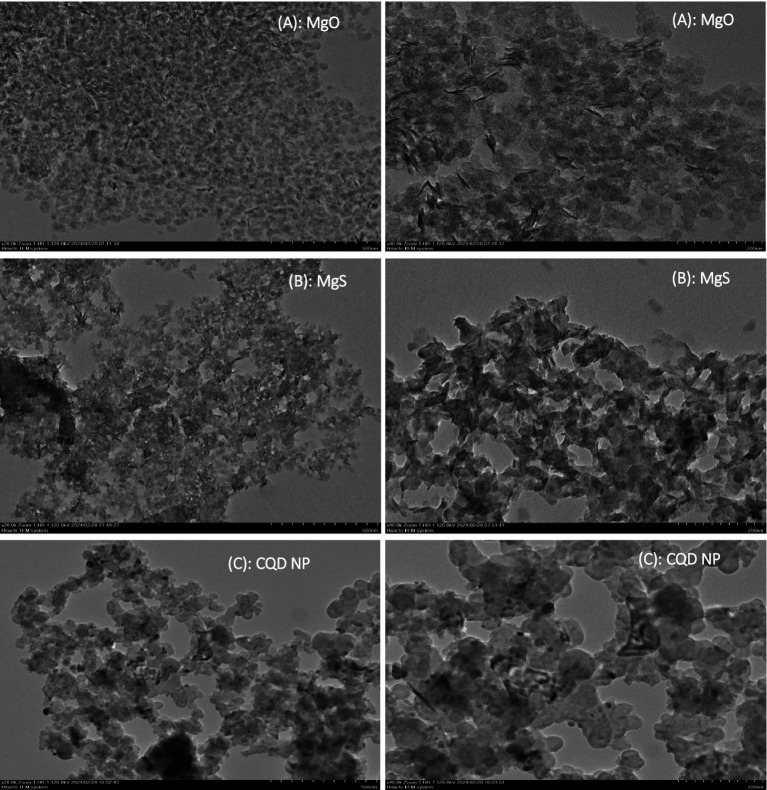
TEM images of **(A)**: MgO NP, **(B)**: MgS and **(C)**: CQD NPs.

The characterisation of MgO NP production using pomegranate extract is depicted in Figure 5A, which is an image captured using a transmission electron microscope (TEM). Here, the scale bars are 500 and 200 nm. The images of TEM analysis of MgS NPs were taken and show the structures of MgS NPs (Figure 5B). The shape of these NPs was a small layer formation with a nearly spherical arrangement on a smooth surface. They had diameters ranging from 20–60 ± 1.6 nm and an average diameter of 55 ± 3.8 nm.

#### Chemical composition

The nutrient composition and relative feed value of algal at various concentrations are indicated in Table 1. The extracts impacted significantly the chemical compositions (all *p* < 0.0001). Regarding fiber fractions such as ADF and NDF, the highest values were recorded for Algae +100 MgS. In contrast, in related to the CP and EE fractions, Algae +50 Mgs had the highest values (*p* < 0.0001).

**Table 1:** Chemical nutrient composition and relative feed value of increasing doses of Algae at different levels of nanoparticles.

Items	Treatment							SEM	*p*-value
	Algae Control	Algae +50 Carbon	Algae +100 Carbon	Algae +50 Mgo	Algae +100 Mgo	Algae +50 MgS	Algae +100 MgS		
CP, %	34.17^ab^	34.68^ab^	32.65^b^	35.98^ab^	32.25^b^	37.23^a^	37.08^a^	1.49	<0.0001
DM, %	95.26^ab^	95.86^ab^	93.31^b^	95.97^ab^	96.80^a^	95.22^ab^	94.69^ab^	0.86	<0.0001
Ash, %	28.40^abc^	29.69^a^	29.43^ab^	27.87^bc^	28.77^abc^	27.46^c^	28.51^abc^	0.59	<0.0001
EE, %	1.25^b^	1.78^b^	1.42^b^	1.17^b^	1.48^b^	2.82^a^	1.68^b^	0.31	<0.0001
NDF, %	31.98^abc^	32.92^ab^	32.67^ab^	33.28^ab^	28.94^c^	30.09^bc^	34.15^a^	1.22	<0.0001
ADF, %	21.79^a^	20.97^ab^	21.45^ab^	21.90^ab^	20.96^ab^	20.62^b^	21.04^ab^	0.38	<0.0001
ADL, %	11.54	11.69	11.88	11.96	10.57	10.40	12.14	0.73	0.069

CP, Crude protein; DM, Dry matter; Ash, ash; EE, Ether extract; NDF, Neutral detergent fiber; ADF, Acid detergent fiber; ADL, Acid detergent lignin. a-c, means within the column with unlike superscript differ significantly (*P* < 0.01).

#### *In vitro* fermentation and gas production

The effects of algae extracts on *in vitro* rumen fermentation profiles are shown in Table 2. The parameters of gas production, pH, and OMD have influenced significantly by the different extracts (all *p* < 0.0001); the highest and lowest values regarding net gas, pH, OMD, ME, and NE_l_ were observed for Algae +50 MgO and Algae +50 MgS, respectively. The converse mentioned trend was observed for Algae +50 MgO and Algae +50 MgS in related to CH_4_ production (*p* < 0.0001). Totally, not only measured total gas volume but also most of the measured parameters from the Algae-based rumen fluid were significantly influenced by the different nanoparticles (*p* < 0.0001).

**Table 2:** Effects of nanoparticles on *in vitro* gas, methane production quantities, and rumen fermentation variables of algae.

Item	Treatment								
	Algae Control	Algae +50 Carbon	Algae +100 Carbon	Algae +50 MgO	Algae +100 MgO	Algae +50 MgS	Algae +100 MgS	SEM	*p*-value
pH	6.77	6.79	6.76	6.78	6.77	6.75	6.79	0.02	0.074
CH_4_, %	18.18^bc^	17.14^bc^	19.12^bc^	16.11^c^	20.77^b^	28.98^a^	25.93^ab^	1.23	<0.0001
CH_4_, mL	5.87	5.27	6.09	5.11	5.92	6.30	5.71	0.57	0.152
Gas, mL	32.32^a^	30.64^a^	31.67^a^	32.04^a^	28.59^a^	21.81^b^	21.99^b^	1.94	<0.0001
TDMA, mg	364.7	389.17	355.83	370.2	395.4	390.41	365.88	13.52	2.672
ME, mj/kg KM	5.91^ab^	5.85^ab^	5.79^ab^	5.99^a^	5.59^ab^	5.53^b^	5.52^b^	0.14	<0.0001
MPSE, mg	83.10^c^	84.88^ab^	83.69^b^	83.36^b^	86.45^ab^	89.29^a^	88.75^a^	1.04	<0.0001
NEL, mj/kg KM	3.19	3.21	3.10	3.26	2.96	3.09	2.97	0.11	0.065
OMD, %	30.26^a^	29.81^a^	30.01^a^	30.23^a^	28.91^a^	26.82^b^	26.94^b^	0.66	<0.0001
PF, mg/mL	364.7	389.17	355.83	370.2	395.4	390.41	365.89	13.52	1.281
TDD, %	70.25^b^	75.00^ab^	69.86^b^	71.23^b^	78.89^a^	75.98^ab^	71.48^b^	2.43	<0.0001

a–c, means within the column with unlike superscript differ significantly (*p* < 0.05). TDMA, true digested matter amount; ME, metabolizable energy; MPSE, Microbial protein synthesis efficiency; NEL, net energy lactation; OMD, organic matter digestion; PF, Partition factor; TDD, true digestion degree; SEM, standard error of means.

#### VFA parameters

Table 2 shows the volatile fatty acid (VFA) composition of rumen fluid. The effects of extracts on total VFA (TVFA) were substantial (*p* < 0.001), with the highest value found in the “Algae +100 MgS” group (163.12 mM) and the lowest in the CON group (139.59 mM). For the individual VFA, extracts have resulted in fluctuated amounts between treatments, in which the treatments influenced the individual VFA significantly (*p* < 0.001).

## Discussion

### Characterization of CQD, MgS, and MgO nanoparticles

#### XRD analysis

**Figure 6:**
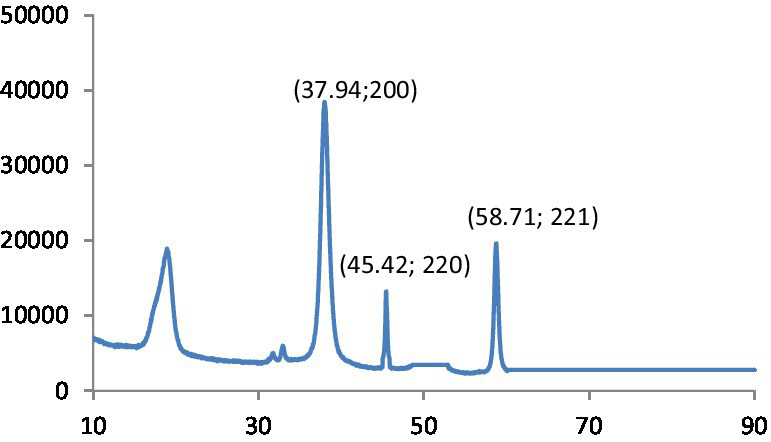
XRD patterns of MgS.

The observed peaks demonstrate the cubic structure of MgO and assign it to the pure phase of periclase MgO. In the spectra of other phases, no additional peaks could be seen. It confirmed that the prepared MgO was crystallized and was free of impurities. In addition, the presented peaks exhibit higher intensity and narrower spectral widths, indicating the product is in good condition. The XRD graph obtained for the crystallographic analysis of synthesized MgS nanomaterials is given in Figure 6. The 2θ values for MgS NPs peak at 37.94° (200), 45.42 (220) and 58.71° (221) at 200, 210 and 222. The characteristic peaks of the XRD spectrum at 2θ = 45.45° can be indexed at (220). Literature-based findings are consistent with the results obtained (24).

#### FTIR analysis

**Figure 7:**
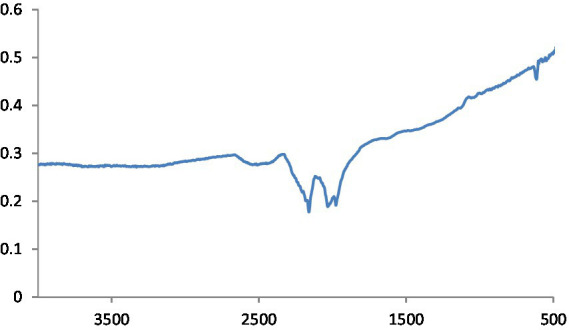
FTIR spectra of CQD NPs.

This is defined as OH stretching and bending, respectively. The metal-oxygen frequencies for the respective metal oxides published in the literature and observed frequencies coincide reasonably well. Using this method, MgO NPs can be analyzed for their chemical composition and surface properties (25). The -C-H bending vibrations in the aromatic amine groups of the flavonoid structure are linked to the absorption band at 550 cm^−1^. Additionally, the peak at 972 cm^−1^ shows the existence of MgS NPs as well as the distinctive C-S bond structure peaks. Under the aliphatic chain structure, the observed 613.4 cm-1 peak is part of the –CH2 group. The pomegranate algae extract’s bioactive components were verified using FTIR spectrum (26). Using this analysis, it is possible to determine the biomolecules involved in the synthesis of MgS NP. Figure 7 displays the carbon quantum dots of NP according to FTIR spectra. The band at around 3,242 cm^−1^ is indicative of OH stretching vibration, which may arise from either the hydroxyl groups found in C black NP or water absorption. The peak recorded at 1,652 cm^−1^ was exclusively found in pure CB and was ascribed to the material’s C=C stretching vibration. Peaks at 2,040, 2,166, and 2,015 cm^−1^ are ascribed to the nanocarbon structure’s carbonyl group and C–O stretching (27).

#### TEM analysis

TEM analysis is a very critical methodology to describe the particle size distribution, average particle size and shape of NPs. The produced MgO nanoparticles are less than 10 nm in size and spherical, as confirmed by TEM examination, despite being aggregated (28). The current green synthesis method approach has enabled the use of a simple and low-cost reducing agent for single-phase MgS NPs. This approach offers an effective method to synthesize MgS NPs in a non-toxic manner (24). Transmission electron microscopy (TEM) evaluated the morphology of pure carbon NP samples. These NPs are the samples showing the highest level of modification and are shown in Figure 5C. The TEM image shows semi-spherical primary particles with an average size ranging from 15 to 65 nm. These primary particles were formed and held together by agglomeration, resulting in agglomerates. The results corroborate other published studies in the literature, and the conclusions are consistent with current literature (29).

#### Chemical composition

The present results on the chemical composition of macroalgae were in line with previous reports (30, 31). In disagreement with our findings, a recent meta-analysis of 47 published papers containing a broad variety of macroalgae was conducted and demonstrated that the average content of CP, NDF, ADF, and organic matter (OM) was 734.2, 189.2, 321.3, and 208.5 g/kg DM, respectively (32). Additionally, to confirm our findings Min et al. (33) published the different levels of CP (7.8 to 38.1% DM), NDF (16.6 to 43.1% DM), ADF (6.6 to 13.1% DM), and EE (0.3 to 3.9% DM) across eight macroalgae species. It’s important to note that the chemical composition and bioactive content of macroalgae are impacted by their taxonomic classification (brown, green, or red), and vary across genera and species. Seasonal fluctuations may also impact their composition during the growing and harvesting periods (31, 34). All algae and nanoparticles tested in our study had acceptable chemical compositions, particularly as a protein source; however, they should be included in a TMR ration to determine their potential advantages.

The current study’s findings about NDF and ADF were congruent with those of Mahmood Ameen (35). Feeds typically comprise 100–120 g/kg DM of ash. The crude ash levels of the feeds were comparable to those reported by Kamalak et al. (36) and Karabulut et al. (37). Differences in nutrient composition of feeds between studies may be qualified to numerous elements, such as climate, fertilization, species and type, harvesting time, feed storage conditions, and vegetative phase (36, 38). Also, it has been stated that the *in vitro* gas production level is affected by the nutrient composition of feedstuff, the presence of compounds inhibiting (such as tannins) gas production, the microflora and microfauna content of the rumen fluid (donor animal’s diet), and the quality of fermentation provided (36, 38).

Microalgae have mass balances ranging from 630 to 1,170 g kg^−1^, although proximate analysis seldom provides 100% (39). Our investigation’s findings regarding the mass balance deficit suggest that other soluble components such as B vitamins, nonprotein nitrogen, chlorophyll, and soluble carbohydrates may be responsible. Microalgae fiber is low in hemicellulose and lacks lignin, even though it has a high fiber content (50–55% of total carbohydrate) (40). This enhances the probability that the protein will be readily available due to its lack of lignin complexation. In addition, the cell wall fraction in microalgae is highly digestible (41). Drewery et al. (42) found that supplementing post-extraction algal residue (CP = 179 g kg^−1^ DM) increased OM digestibility in steers fed oat straw (CP; 45 g kg^−1^ DM). Similarly, Tetracystis sp., *N. bacillaris*, and *C. vulgaris* have a higher lipid content, which improves the calorie density of the diet. It has been widely shown that lipids frequently diminish enteric CH_4_ emissions from ruminants (43, 44).

#### *In vitro* fermentation and gas production

The post-fermentation pH ranged from 6.75 to 7.79 among algal-extract treatments, demonstrating that algae supplementation promotes a more alkaline environment during microbial fermentation. Carbohydrates are the primary source of substrate for the creation of acetate and butyrate during ruminal fermentation, and as byproducts, CO_2_ and hydrogen (H_2_), are used by methanogenic archaea to produce CH4 (9). Furthermore, according to Kholif et al. (45), microalgae promote carbohydrate fermentation by rumen microbes, which is consistent with what was observed with the addition of microalgae and was attributed to the microalgae’s fulvic acids, which can provide carbon to ruminal microorganisms (46) and thus favor microbial growth and increase DMD. In turn, the increased degradability resulted in higher production of SCFA and ME, ascribed to enhanced carbohydrate degradation (45).

Although not investigated in the current study, the increase in SFCA and ME with microalgae might be due to increased activity of the fibrolytic bacteria (47) and increased propionate production. In contrast, decrease of SFCA and ME are attributed to a reduction in other SCFAs, such as acetate (48). In the meantime, the effects on DMD and SCFA associated with the content and degradability of feed carbohydrates may be reflected in the computed variations in CH_4_ per unit of SCFA, ME, and OM (49).

Biogas production (BG) is intimately related to feed degradability and, as a result, the availability of highly-fermented nutrients for rumen microbial activity and growth (15). Although their production is predominantly reliant on the fermentation of carbohydrates to SCFA and proteins, and BG is mostly made up of CO_2_ and CH_4_, their contribution to BG is negligible in comparison to that of carbohydrates (50). Furthermore, the production of acetate and butyrate during rumen fermentation produces more gas than the formation of propionate, accounting for the majority of the BG (51).

Natural compounds of microalgae have been proposed as potential methods for controlling rumen fermentation, contributing to CH_4_ generation (52, 53). A previous *in vitro* investigation (54) demonstrated that Schizochytrium spp. inhibited CH_4_. Furthermore, several research (55, 56) found an increase in CH_4_-producing bacteria and protozoa, demonstrating that not all microalgae have CH_4_-reducing properties.

The anti-methanogenic effect observed in this study has been reported in studies involving other microalgae (Spirulina platensis, *Chlorella vulgaris*, and Schizochytrium spp.). The studies attribute this effect to the presence of docosahexaenoic acid (C22:6 n − 3) and eicosapentaenoic acid (C20:5 n − 3), polyunsaturated acids that decrease the concentration of acetate and increase propionate, which results in reduction the abundance of methanogenic archaea, the primary microorganisms producing CH_4_ (15, 51, 52). Likewise, Sheng et al. (57) found that humic compounds, including fulvic and humic acids, can lower CH_4_ production in ruminants. They ascribed this to a decrease of the molar proportion of protozoa and acetate populations (58), which minimizes the amount of H_2_ available for CH_4_ production (59).

The addition of the microalgae reduced BG production in the current study, which is in line with Elghandour et al. (15), who observed that the BG decreased with the addition of the microalgae Schizochytrium spp. and associated it with the antimicrobial and cytotoxic effects of the compounds of the microalgae (60), as well as the long-chain fatty acid profile (48). Also, it is likely that the microalgae have modified the structure of the microbial community during fermentation which is caused variations in the final fermentation products, including the SCFA profile (61).

Among the treatments, the highest amount of gas produced was observed for the MgS nanoparticles group. Additionally, the MgO treatments demonstrated a notable decrease in the production of methane, which indicate the ability of MgO nanoparticles to meet the needs of rumen bacteria during the incubation period (62). The two main sources of *in vitro* gas generation are carbon dioxide and methane, which are derived directly from microbial fermentation, and carbon dioxide released from a bicarbonate buffer, obtained indirectly by buffering short-chain fatty acids. Menke and Steingass (23) affirm that the only variables influencing gas generation are the feed’s physical and chemical composition. The fermentation rate, however, could be impacted by modifications in ruminal microbial activity.

#### VFA parameters

Volatile fatty acids (VFAs) have been considered one of the most significant factors in achieving anaerobic fermentation. According to Makkar (63), fluctuations in gas production might alter the amounts or ratios of VFA produced. VFAs’ hydrophobic qualities enable them to penetrate the bilayer structure of the bacterial cell’s plasma membrane (64). Therefore, by changing the membrane structure and increasing its flowability and permeability, they can lower the rate of bacterial growth (65).

Previous research has shown that adding red algae (*Asparagopsis taxiformis*) and lipid-extracted microalgae to forage diets dramatically boosted propionate and butyrate levels in the rumen (66). This is deemed advantageous since previous research demonstrated that the energy from propionate was used more efficiently than energy from acetate (67, 68). Lodge-Ivey et al. (68) found that adding lipid-extracted algae (Chlorella or Nannochloropsis) to the diet increased total rumen VFA content, which is in consistent with our findings. In contrast to our findings, it has been proposed that the high lipid content of Chlorella may suppress cellulolytic bacteria in the rumen and finally reduction of total VFA (53). Furthermore, adding algae to a corn silage-based diet raised ruminal pH and reduced total VFA by up to 18% after 19 days (69). Other *in vitro* investigations (53–55, 70) found that supplementation with DHA-rich microalgae or marine algae increased ruminal propionate while decreasing overall VFA and CH_4_ synthesis. The differences could be attributed to variances in supplementation levels and oil extraction.

## Conclusion

The results of our study indicate that the use of Algae+50 Mgo nano-particles, viable feed additive, highest in CP and EE, can reduce methane emission and gas production. Furthermore, all the treatments containing Algae decreased *in vitro* gas production. Also, addition of the Algae+50 Mgo nano-particles improved fermentation kinetics, VFAs, and nutrients’ degradability compared to the other experimental treatments. These results are promising and suggest that the applied extracts could mitigate undesirable outcomes of rumen fermentation. Although more research is necessary to clarify the exact effects of the extracts on the aforementioned indices.

## Data Availability

The original contributions presented in the study are included in the article/supplementary material, further inquiries can be directed to the corresponding author/s.
